# Sequential Kinase Inhibition (Idelalisib/Ibrutinib) Induces Clinical Remission in B-Cell Prolymphocytic Leukemia Harboring a 17p Deletion

**DOI:** 10.1155/2017/8563218

**Published:** 2017-07-27

**Authors:** H. Coelho, M. Badior, T. Melo

**Affiliations:** Serviço de Hematologia, Centro Hospitalar de Vila Nova de Gaia/Espinho, Vila Nova Gaia, Portugal

## Abstract

B-cell prolymphocytic leukemia (B-PLL) is a rare lymphoid neoplasm with an aggressive clinical course. Treatment strategies for B-PLL remain to be established, and, until recently, alemtuzumab was the only effective therapeutic option in patients harboring 17p deletions. Herein, we describe, for the first time, a case of B-cell prolymphocytic leukemia harboring a 17p deletion in a 48-year-old man that was successfully treated sequentially with idelalisib-rituximab/ibrutinib followed by allogeneic hematopoietic stem cell transplant (allo-HSCT). After 5 months of therapy with idelalisib-rituximab, clinical remission was achieved, but the development of severe diarrhea led to its discontinuation. Subsequently, the patient was treated for 2 months with ibrutinib and the quality of the response was maintained with no severe adverse effects reported. A reduced-intensity conditioning allo-HSCT from a HLA-matched unrelated donor was performed, and, thereafter, the patient has been in complete remission for 10 months now. In conclusion, given the poor prognosis of B-PLL and the lack of effective treatment modalities, the findings here suggest that both ibrutinib and idelalisib should be considered as upfront therapy of B-PLL and as a bridge to allo-HSCT.

## 1. Introduction

B-cell prolymphocytic leukemia (B-PLL) is a rare lymphoid neoplasm constituting approximately 1% of all cases of lymphocytic leukemia [1]. The median age at diagnosis of B-PLL is 69 years, and the condition has a similar distribution pattern among male and female patients, who typically present with B symptoms, marked lymphocytosis, massive splenomegaly, and minimal lymphadenopathy [2]. The diagnosis requires the presence of prolymphocytes, exceeding 55% of lymphoid cells in the peripheral blood [3]. However, distinguishing between B-PLL and chronic lymphocytic leukemia (CLL)—which has increased number of prolymphocytes and the blastoid variants of mantle cell lymphoma—solely on the basis of morphological assessments may be difficult, necessitating usage of immunophenotypic studies [3, 4]. B-PLL cells strongly express the surface immunoglobulins, IgM+/−IgD, and various B-cell antigens (CD19, CD20, CD22, CD79a and CD79b, and FMC7). Positive CD5 and CD23 expressions are seen in only 20–30% and 10–20% of the cases, respectively [2].

Treatment strategies for B-PLL remain to be established. The largest clinical trial of B-PLL included only 14 patients treated with pentostatin, and all other studies are limited to case reports or series with lesser than 10 patients [5]. Therefore, in the absence of clinical trials, clinicians typically employ a CLL-like treatment approach. Combinations of rituximab with fludarabine or bendamustine together with an anthracycline (mitoxantrone or epirubicin) have been reported to show significant activity in B-PLL [6–8]. However, resistance to purine analog/alkylator based therapy is high among B-PLL patients, as more than half of these patients have* TP53* abnormalities including del(17p) [3, 9, 10]. Until recently, alemtuzumab was the only effective therapeutic option for these patients, despite being associated with high rates of infections and short-lived responses [11, 12]. Lately, ibrutinib or idelalisib combined with rituximab has shown the best treatment outcome (response rates, progression-free survival, and overall survival) in* TP53*-disrupted CLL patients [13, 14]. Nevertheless, little is known about the treatment outcome of these new drugs in B-PLL, as very few patients have received either ibrutinib or idelalisib-rituximab [15, 16].

Herein, we report, for the first time, the efficacy of sequential kinase inhibition therapy (idelalisib-rituximab/ibrutinib) followed by allo-HSCT, in a patient diagnosed with B-PLL.

## 2. Case Presentation

**Figure 1:**
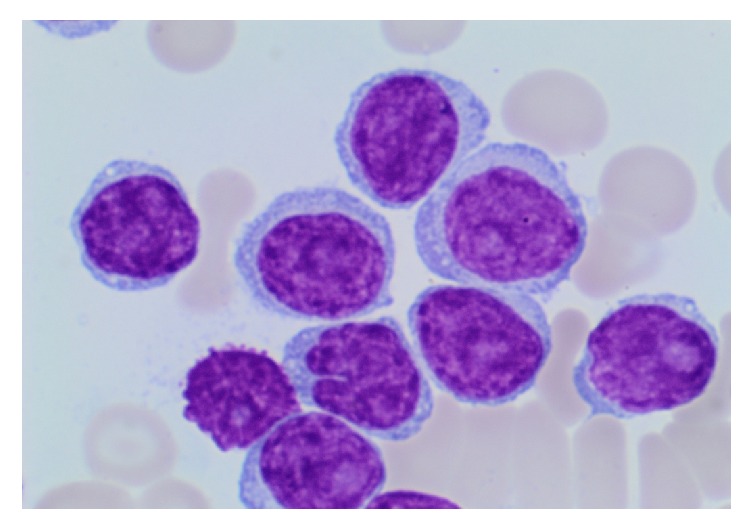
Peripheral blood smear. The lymphoid cells are of intermediate to large size with a prominent nucleolus and cytoplasm protrusions (Wright-Giemsa, ×1000).

A 48-year-old man was admitted in July 2015 with complains of abdominal discomfort, progressive night sweats, and a 10-kg weight loss over a period of 6 months. A full blood count showed the following findings: white blood cell count, 622 × 10^9^/L; hemoglobin (Hb) concentration, 80 g/L; platelet count, 83 × 10^9^/L; and LDH, 378 IU/L. A peripheral blood film demonstrated marked lymphocytosis with variably sized lymphocytes having clumped chromatin and central nucleoli (Figure 1). Peripheral blood immunophenotyping demonstrated that the lymphocyte population was composed almost exclusively of monoclonal B cells (95% of the lymphoid cells) with the following immunophenotype: CD19^+^, CD20^+^, CD79b^+^, CD5^+^, FMC7^+^, CD23^+/−^, and CD10^−^. Fluorescent in situ hybridization (FISH) showed positive findings for del(17p) in 86% of the cells and negative findings for deletion of 11q, 13q, trisomy 12, and t(11; 14). A bone marrow biopsy revealed that B-PLL accounted for 80% of the cellularity in a markedly hypercellular marrow. The bone marrow was diffusely infiltrated with lymphoid cells expressing CD5, CD20, and CD79a, whereas CD10-, CD21-, CD23-, BCL6-, Cyclin D1-, and SOX11-positive cells were absent. A computed tomography scan of the chest, abdomen, and pelvis showed the presence of hepatomegaly and splenomegaly (craniocaudal height, 23 cm).

**Figure 2:**
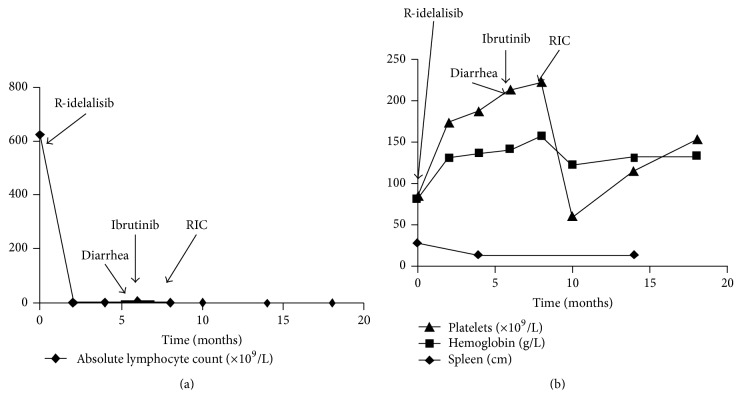
Response to idelalisib-rituximab started on day 0, ibrutinib started in month 6, and reduced-intensity conditioning allo-HSCT was performed in month 8. (a) Absolute lymphocyte count. (b) Platelet count, hemoglobin concentration, and spleen size (measured by CT scan/longest diameter).

The patient started receiving idelalisib (150 mg twice daily) and rituximab (375 mg/m^2^; every two weeks for five doses and then every four weeks for 3 doses). The first two administration instances of rituximab were omitted due to a high tumor burden. Treatment with idelalisib-rituximab led to a rapid resolution (within 2 months) of the lymphocytosis (3.8 × 10^9^/L), accompanied by normalization of the Hb concentration (130 g/L) and platelet count (174 × 10^9^/L) and resolution of the hepatosplenomegaly on CT (Figure 2). After 5 months of therapy, the patient developed grade 3 diarrhea (National Cancer Institute Common Terminology Criteria for Adverse Events v4.0) that resolved after treatment with prednisone (1 mg/kg id) along with discontinuation of idelalisib for 1 month. Thereafter, idelalisib was restarted at a low dose (150 mg daily), but recurrence of the diarrhea during the first week of treatment led to a definitive suspension and a change in therapy. Ibrutinib (420 mg daily) was commenced 2 weeks after idelalisib interruption, and, within the first 2 weeks, the patient developed a transient mild lymphocytosis (15 × 10^9^/L). However, no significant side effects or clinical signs of B-PLL progression were noted, and, after a period of 2 months with ibrutinib, the Hb concentration (156 g/L), lymphocytes (4 × 10^9^/L), and platelets (222 × 10^9^/L) were found to be within the normal range, with no spleen enlargement on physical examination. The patient was treated with ibrutinib until admission for bone marrow transplantation. A reduced-intensity conditioning (RIC) allo-HSCT from a 10/10 HLA-matched unrelated donor was performed. The conditioning regimen included fludarabine (30 mg/m^2^ for 6 consecutive days; days −10 to −5), oral busulfan (4 mg/kg/d for 2 consecutive days; days −6 to −5), and anti-T-lymphocyte globulin (10 mg/kg/d for 4 consecutive days; days −4 to −1) as well as prophylaxis against graft-versus-host disease (GvHD) with mycophenolate mofetil and tacrolimus tapered over 7 months. The regimen was well-tolerated with very mild toxicity and no major transplant-related complications. Six months after the transplant, counts of the lymphocytes, the neutrophils, and platelets and also the Hb concentration were within the normal range and no enlarged organs were detected on CT (Figure 2). In addition, flow cytometry and immunohistochemistry revealed that the bone marrow was free of clonal lymphoid cells. Furthermore, the FISH test carried out to detect leukemic cells harboring a 17p deletion yielded negative results. The patient has been well and in complete remission for 10 months now.

## 3. Discussion

B-PLL treatment modalities remain to be well-established and little is known about the efficacy of ibrutinib or idelalisib-rituximab for this disease. Recently, 5 patients with B-PLL carrying a genetic disruption of* TP53* were treated with idelalisib, and sustained remissions (6–10.5 months) were observed in 3 of these patients [15]. Adverse events were noted, namely, grade 2-3 transaminitis (3 patients) and CMV reactivation (1 patient), leading to temporary treatment interruption [15]. Ibrutinib was administered to 2 patients with B-PLL harboring complex cytogenetic abnormalities with aberrations in the* TP53* gene. Disease control was achieved with lymphocytosis resolution, accompanied by an improvement in Hb level, platelet count, and splenomegaly. Contrary to CLL, an increase in lymphocytosis was not observed following administration of ibrutinib [16].

Because of the availability of new promising alternative drugs, allogeneic hematopoietic stem cell transplantation (allo-HSCT), regarded as the only curative option for CLL, is now frequently deferred [17]. However, as B-PLL is often characterized by high-risk genetic abnormalities, which partly explains the poor outcomes and short survival times associated with conventional chemoimmunotherapy, suitable patient candidates should be considered for allo-HSCT on first remission.

In the present case, a rapid and meaningful response was observed in* TP53*-disrupted B-PLL treated sequentially with idelalisib-rituximab and ibrutinib. The response with idelalisib-rituximab was relatively robust and a toxicity profile similar to that already described in CLL was observed. As infusion-related reactions can be markedly decreased if the tumor load is first reduced with an initial course of rituximab-free chemotherapy, 2 cycles of rituximab were omitted [18]. Severe diarrhea, which developed in our patient, is one of the most common adverse events associated with idelalisib (7% of the patients) that prompts dose reduction (34%) and treatment discontinuation (20%) [19]. The clinical presentation of grade 3 diarrhea, after 5 months of therapy with idelalisib, as well as the time to its resolution (interruption of idelalisib and initiation of corticosteroid treatment) was as expected, concurring with another published report [19]. After having suspended idelalisib treatment, our patient started receiving ibrutinib. The disease remained in remission and no adverse events were registered. Ibrutinib is known to promote high response rates, leading to durable remissions in all genetic subsets of CLL patients, and discontinuation of ibrutinib is rarely due to adverse events related to the drug [20, 21]. Interestingly, we have observed an increase in lymphocytosis following the administration of ibrutinib, similar to that seen in CLL. The lymphocytosis, however, does not appear to be associated with an increased risk of progression; nonetheless, these circulating cells may go back to tissue, and disease may become more aggressive following discontinuation of ibrutinib [21].

## 4. Conclusion

In conclusion, given the poor prognosis of B-PLL and the lack of effective treatment modalities, the findings presented here suggest that both ibrutinib and idelalisib should be considered as upfront therapeutic options for B-PLL and as a bridge to allo-HSCT in eligible patients.
